# The Effects of Strength, Plyometric and Combined Training on Strength, Power and Speed Characteristics in High-Level, Highly Trained Male Youth Soccer Players: A Systematic Review and Meta-Analysis

**DOI:** 10.1007/s40279-023-01944-8

**Published:** 2023-10-28

**Authors:** Jon L. Oliver, Akhilesh Kumar Ramachandran, Utkarsh Singh, Rodrigo Ramirez-Campillo, Rhodri S. Lloyd

**Affiliations:** 1 Youth Physical Development Centre, Cardiff School of Sport and Health Sciences, Cardiff Metropolitan University, Cardiff, CF23 6XD UK; 2 Sport Performance Research Institute New Zealand (SPRINZ), Auckland University of Technology, Auckland, New Zealand; 3 Sports and Exercise Science, College of Healthcare Sciences, James Cook University, Townsville, QLD 4811 Australia; 4 Faculty of Rehabilitation Sciences, Exercise and Rehabilitation Sciences Institute, School of Physical Therapy, Universidad Andres Bello, 7591538 Santiago, Chile

## Abstract

**Background:**

Male youth soccer players competing at a high level will typically engage in large volumes of soccer training from a young age. However, it is not known whether the high levels of habitual training that these high-performing players are exposed to limit their ability to respond to strength, plyometric or combined training interventions.

**Objective:**

The primary aim of our systematic review and meta-analysis was to compare the specific effects of strength, plyometric and combined training with active controls (standard soccer training) on the strength, power and speed characteristics of high-level, highly trained young male soccer players.

**Methods:**

We performed a literature search across PubMed, Scopus, CINAHL, Web of Science and SPORTDiscus to identify controlled studies that implemented strength, plyometric or combined training in high-level male youth soccer players. Participants were defined as high level or highly trained based on established guidelines related to either competition level or age-related weekly hours spent in soccer training. Studies needed to report at least one outcome of lower body strength, squat jump, countermovement jump, horizontal power, acceleration (0–10 m), speed (15–40 m) or change of direction speed. A meta-analysis was then performed using a random-effects model to determine the magnitude (Hedge’s g) of training responses and whether effects differed across modes of training.

**Results:**

From an initial return of 5464 papers, *n* = 34 studies met the inclusion criteria and provided a total sample of *n* = 1396 high-level male youth soccer players. Strength, plyometric and combined training resulted in improvements in strength, squat and countermovement jump, horizontal power, acceleration, change of direction speed (all* p* < 0.05; *g* = 0.73–1.08, moderate) and speed (*p* < 0.05; *g* = 0.40–0.59, small). Lower body strength was the only outcome where training mode had a significant effect (*p* < 0.05), with plyometric training producing small effects (*g* = 0.27, *p* < 0.05) compared with moderate effects for strength (*g* = 1.07, *p* < 0.05) and combined (*g* = 0.75, *p* < 0.05) training. Prediction intervals for overall effects (all training modes combined) showed that the greatest confidence that future training will lead to positive effects was in the squat and countermovement jump, horizontal power and acceleration (prediction intervals = 0.03–1.81).

**Conclusions:**

High-level, highly trained male youth soccer players can experience positive gains in indices of strength, power and speed from strength, plyometric and combined training, and the magnitude of gains are mostly similar across modes of training. Based on prediction intervals, there is a good level of certainty that future strength, plyometric and combined training in this population would lead to positive improvements in vertical and horizontal power and sprint acceleration.

**Supplementary Information:**

The online version contains supplementary material available at 10.1007/s40279-023-01944-8.

## Key Points

**Table:** 

Despite engaging in high volumes of systematic soccer training from a young age, high-level, highly trained male youth soccer players can still make positive improvements in strength, power and speed characteristics following strength, plyometric and combined training interventions.
Strength gains in high-level, highly trained male youth soccer players are greater following strength or combined training when compared with plyometric training.
It is likely that future strength, plyometric or combined training in high-level, highly trained male youth soccer players will lead to positive improvements in squat and countermovement jump performance, horizontal power and acceleration.
While all forms of training led to mostly moderate improvements in strength, jump performance, acceleration and change of direction speed, maximal speed only improved by a small amount.

## Introduction

Soccer is considered to be highly physical and demanding in nature [1, 2], even at a youth level [3–6]. During a full-length game, players can be required to perform ~ 1350 high-intensity activities, including jumps, brief sprints and changes of direction involving rapid accelerations and decelerations [7]. These strength, power and speed-related actions are important for success, contributing towards retaining or retrieving ball possession and creating goal scoring opportunities during match play [8–10]. The ability to produce high levels of sprint speed, change of direction speed (CODS) and jump performance have also been shown to distinguish between more and less successful players at the junior level [10–12]. Therefore, player development pathways may consider it important to develop strength, power and speed characteristics from a young age.

Soccer has been suggested to be moving towards an approach of early specialisation with high training volumes in the academy setting [13]. Data from a multitude of countries across the Americas, Europe and Africa show that professional soccer players start playing the game at around 5 years of age and begin specialising between the age of 10 and 12 years [14–16]. By the time high-level young players (e.g. those in academies and representative teams) reach the U16 age group, they can have accumulated 3900–5500 h of training, with adolescent players training and playing soccer for an average of 12 h per week [15], reflecting the fact they are highly trained. Most of the training load that young, highly trained soccer players will be exposed to is the format of field-based training (e.g. technical training, small-sided games) and match play [17]. The observation that elite young soccer players are stronger, can sprint faster, change direction quicker and jump higher than their non-elite counterparts [10, 12, 18, 19] may reflect a selection bias towards players who demonstrate a high level of physical fitness. Alternatively, the physical superiority of young players competing at higher levels may reflect a positive adaptation to being highly trained following exposure to a high volume of specific systematic training. For instance, over a period of multiple years, the ability to jump, sprint and change direction improves more in academy soccer players compared with those who only play for their school team [20].

While physical fitness may improve in young soccer players as a result of involvement in soccer-specific training, resistance training has the potential to provide an additional stimulus that can aid long-term athletic development [21, 22]. Resistance training is considered a specialised form of training where individuals work against resistance provided by body weight or an external load in the form of free weights, medicine balls, elastic bands or weight machines [23]. Strength and plyometric training are common forms of resistance training used to improve the athletic abilities of young athletes [21]. For soccer, enhancing strength may be useful as strength has been shown to be related to acceleration, speed, CODS and power in youth soccer players [24–28]. Similarly, plyometric training has become a popular training method in youth soccer [29, 30], with observations that this form of training can improve muscular power, maximal strength, and sprinting and acceleration capabilities in young male and female soccer players [31–33]. Given different forms of training will stimulate different adaptations, practitioners and researchers have looked to combine different forms of training (e.g. strength and plyometric training) to maximise performance responses, reporting strength, power and speed improvements with this approach in young soccer players [34–36].

It is now widely accepted that resistance training can have a positive effect on the physical development of children and adolescents [21], with the positive responses of youth to resistance training well documented in a number of meta-analyses [37–44]. However, these meta-analyses consider either a general population of children and adolescents or populations of youth athletes across a mix of different sports and abilities. Bedoya and Miltenberger [45] have provided a review of plyometric training in youth soccer players, although this review covered players of all standards and did not provide any meta-analysis. Similarly, a previous meta-analysis of the effect of plyometric training in “young” soccer players ignored training status and also extended to players in the U23 age group [33], combining results for immature and fully mature players. High-level, highly trained young soccer players represent a unique population as they are exposed to a high volume of training, which will include a high exposure to sprinting, accelerating and decelerating, changing direction and jumping. The high level of exposure to explosive actions during soccer training and play may then limit the ability of youth soccer players to further adapt these qualities following resistance training, with review work demonstrating that trained youth athletes respond. Both Lesinski et al. [41] and Behm et al. [39] have shown that responses to resistance training in youth are specific to the population, type of training and outcome. For instance, Behm et al. [39] showed that trained youth athletes experience smaller gains in strength and power than their untrained counterparts, attributing this response to differences in baseline fitness and training history. Behm et al. also demonstrated the effect of training specificity with strength training having a much greater effect on strength than on jump performance, and power training having a much greater effect on jump performance than strength. To the authors knowledge, no existing meta-analyses have considered the specific effects of different forms of resistance training in high-level, highly trained youth athletes from a single sport, athletes who will have already been exposed to a high volume of systematic training and will likely have high levels of baseline fitness. Therefore, the purpose of this systematic review and meta-analysis was to examine the specific effects of strength, plyometric and combined training on the strength, power and speed characteristics of high-level, highly trained male youth soccer players.

## Methods

### Registration and Literature Search

This meta-analytical review was conducted in accordance with the Preferred Reporting Items for Systematic Review and Meta-Analyses guidelines [46]. The study was registered in Open Science Framework (OSF Registries), an open-source online tool developed by the Centre of Open Science. The registered document can be accessed using the following link: https://osf.io/brmk9.

Literature searches were conducted in the following electronic databases from inception to June 2022 without any restriction to language or publication status: PubMed, Scopus, CINAHL, Web of Science and SPORTDiscus. Publications not presented in English were then removed as part of the screening process of inclusion and exclusion criteria. Keywords were collected through experts’ opinion, a systematic literature review and controlled vocabulary (e.g. Medical Subject Headings: MeSH). A Boolean search was conducted in each database using terms including “youth” OR “child*” OR “young” OR “puberty*” AND “soccer” OR “football” AND “training” OR “Intervention” AND “strength” OR “plyometric” OR “combined” OR “Jump” OR “explosive” OR “ballistic”. Specific search details for each database are provided in the Electronic Supplementary Material (ESM). The search was conducted using a publication date up until the end of June 2022. In addition to the main electronic systematic searches, reference lists of articles that satisfied the inclusion and exclusion criteria were also reviewed.

### Inclusion Criteria

Two of the authors (AR and US) conducted the initial search, removed duplicates and then screened papers against the inclusion criteria. Where there was any disagreement, a third author (JLO) was included in the review process to provide a consensus. A PICOS (participants, intervention, comparators, outcomes and study design) approach was used to rate studies for eligibility [47]. The respective inclusion/exclusion criteria adopted in our meta-analysis were as follows.

#### Population

High-level, highly trained male soccer players competing in U18 age groups and below. High-level or highly trained was defined using one of the two below-mentioned criteria. First, players were categorised as high level based on a competitive standard if they were identified as being Tier 3 athletes or above based on the classification system of McKay et al. [48]. In team sports, Tier 3 athletes include those in academy programmes and who are competing in national or state (regional) level leagues and tournaments. Where the competitive level was not reported, participants were classified as highly trained based on the number of total training hours (team plus individual training) in line with those reported across top-level European Soccer Academies [49]; U12 = 4.5–6 h/week, U13–U15 = 10 h/week, U16–U18 = 10–12 h/week.

#### Intervention

Studies that exposed participants to strength training, plyometric training or combined training for a duration of at least 4 weeks. Combined training included interventions that combined strength or plyometric training with other forms of training (e.g. strength + plyometric, plyometric + sprint).

##### Comparator

Active control group who continued with their normal soccer training.

##### Outcome

At least one measure related to (1) strength; lower body strength, (2) power; squat or countermovement jump or horizontal power (e.g. horizontal jump or bounding) or (3) speed; sprint performance (acceleration or speed) or CODS.

##### Study Design

Controlled trials. Studies in which the use of exercise protocols were not clearly described, studies for which only the abstract was available, case reports, special communications, letters to the editor, invited commentaries, errata, overtraining studies and detraining studies were excluded.

### Methodological Quality and Risk of Bias

The Physiotherapy Evidence Database (PEDro) scale was used to assess the methodological quality of the included studies, which were rated from 0 (lowest quality) to 10 (highest quality). The validity and reliability of the PEDro scale has been established previously [50–52]. Established cut-off scores indicated methodological quality was rated as ‘poor’ (< 4), ‘fair’ (4–5), ‘good’ (6–8) and ‘excellent’ (9–10) [53]. The methodological quality of each included study was assessed independently by two authors (AR and US), and any discrepancies between the two authors were resolved via consensus with a third author (JLO). Risk of bias was evaluated via visual inspection of funnel plots, assessing the level of asymmetry and the inverted funnel shape of the plot. Statistical tests of funnel asymmetry were not employed as these are reported to be under-powered where only a small number of studies are available [54, 55].

### Data Extraction and Interpretation

Descriptive information, including participants’ age, competitive standard and weekly training volume, was extracted where available. Information on the training programme design was recorded, including details on training duration, frequency, intensity, volume (e.g. number of repetitions and number of sets) and recovery. Performance on physical tests evaluating strength, power and speed pre- and post-training was extracted and included measures of lower body strength, squat jump height, countermovement jump height, horizontal power, linear acceleration and speed, and CODS. To prevent the inflation of effects, where a study reported multiple measures for a given outcome (e.g. multiple change of direction tests), a single outcome was extracted and used for analysis. Acceleration was presented as sprint time over distances of 0–10 m, and speed was represented as sprint time over distances of 15–40 m [56]; where multiple distances/split times were reported, the longest distance for each outcome was used (e.g. 10 m and 40 m). Where multiple CODS tests were reported, the test with the longest time to completion was taken forward to the analysis [57]. Horizontal power was represented by jump distance in tests such as a standing broad jump, triple hop and bounding tests; where several outcomes were reported, tests of a single jump effort were used, or where not available, the test with the fewest rebound jump efforts was used for analysis.

In cases where the required data were not clearly or completely reported, the authors of the study were contacted for clarification. If no response was obtained from the authors after two attempts, or if the authors could not provide the requested data, the study outcome was excluded from further analysis. If data were only displayed in the form of figures but not tables, numerical data were extracted from figures using appropriate software (WebPlotDigitizer; https://apps.automeris.io/wpd/). This procedure has proven to be valid (*r* = 0.99, *p* < 0.001) [58].

### Statistical Analysis

Studies were meta-analytically aggregated if three or more relatively homogeneous studies were available for the same outcome measure. Effect sizes (Hedge’s *g*) were calculated by comparing change in performance pre-to-post intervention in training groups relative to changes in control groups for each dependent variable. Data were standardised using post-intervention standard deviation values. The random-effects model was used to account for differences between studies that might influence training effects [59, 60]. Effect size values were presented with 95% confidence intervals (95% CIs) and the magnitude of effects were interpreted using the following scale: < 0.2, trivial; 0.2–0.6, small; > 0.6–1.2, moderate; > 1.2–2.0, large; > 2.0–4.0, very large; > 4.0, extremely large [61]. In studies including more than one intervention group, the sample size of the active control group was proportionately divided to facilitate comparisons across multiple groups [59]. The impact of study heterogeneity was assessed using an *χ*^2^ test and the *I*^2^ statistic, with values of < 25%, 25–75% and > 75% representing low, moderate and high levels, respectively [62]. Heterogeneity and confidence in future effects were further investigated by calculating prediction intervals for each dependent variable [63]. All analyses were carried out using the Comprehensive Meta-Analysis software (Version 2.0; Biostat, Englewood, NJ, USA) with forest plots generated using Review Manager (Version 5.4, Cochrane collaboration, Oxford, UK) based on the results obtained from Comprehensive Meta-Analysis software. The level of statistical significance was set at *p* < 0.05.

## Results

### Study Characteristics

**Fig. 1:**
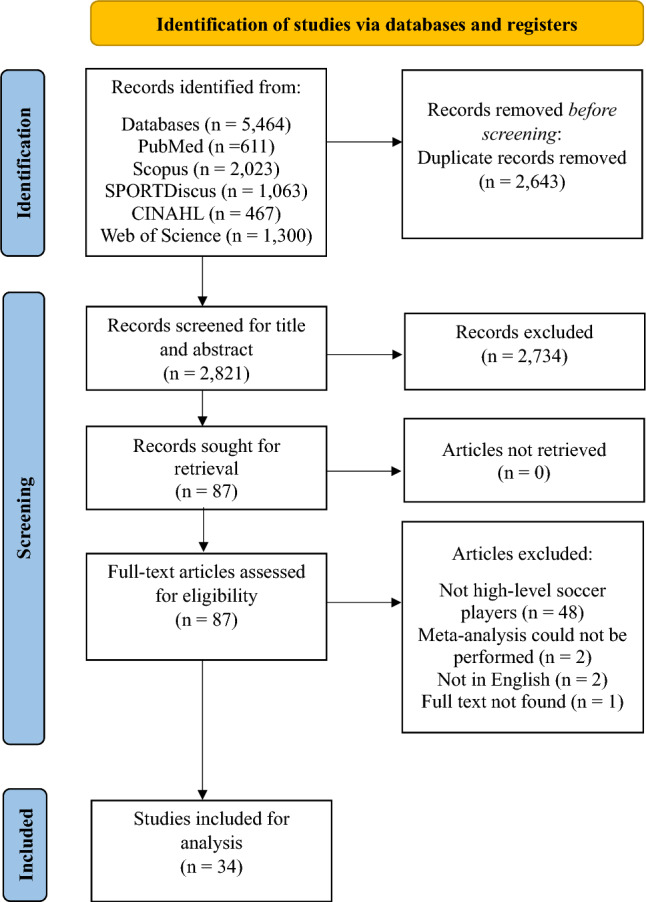
Summary PRISMA (preferred reporting items for systematic reviews and meta-analyses) flowchart identifying the study selection process

The initial database search returned 5464 studies. As shown in Fig. 1, after removing duplicates, screening titles and abstracts, and accessing full-text articles, 34 studies [26, 28, 29, 34, 35, 64–92] were identified as meeting the inclusion criteria. Table 1 provides the PEDro rating of included studies. As expected, no studies were able to blind participants (item 5), only one study claimed to blind those administering an intervention (item 6), while no studies blinded those taking assessments (item 7). Studies scored well for groups being similar at baseline (item 4), outcomes being assessed in at least 85% of participants with participants remaining in the allocated group, statistical outcomes reported with point measures and measures of variability (items 8–11). Overall, one study was rated as poor [34], 12 studies as fair [28, 29, 35, 64, 65, 68, 74–76, 79, 82, 86] and 21 studies [26, 66, 67, 69–73, 77, 78, 80, 81, 83–85, 87–92] rated as having good methodological quality. Funnel plots are presented in the ESM. The plots for lower body strength, squat jump and countermovement jump show no obvious asymmetry, although plots are limited to a clustering of effects around a range of studies with similar sample sizes. For horizontal power, acceleration, speed and CODS, an absence of data points towards the lower left-hand area of the plots suggests an under-representation of smaller studies with limited effects, indicating a bias towards smaller studies with larger effects.

**Table 1:** Methodological quality for included studies using the Physiotherapy Evidence Database (PEDro) rating scale

Study name	1	2	3	4	5	6	7	8	9	10	11	Total^a^	Study quality
Abade et al. [88]	1	1	0	1	0	0	0	1	1	1	1	6	Good
Aloui et al. [89]	1	1	0	1	0	0	0	1	1	1	1	6	Good
Aloui et al. [92]	1	1	0	1	0	0	0	1	1	1	1	6	Good
Aloui et al. [91]	1	1	0	1	0	0	0	1	1	1	1	6	Good
Aloui et al. [90]	1	1	0	1	0	0	0	1	1	1	1	6	Good
Maio Alves et al. [64]	1	0	0	1	0	0	0	1	1	1	1	5	Fair
Boraczynski et al. [65]	1	1	0	0	0	0	0	1	1	1	1	5	Fair
Chelly et al. [26]	1	1	0	1	0	0	0	1	1	1	1	6	Good
Chtara et al. [66]	1	1	0	1	0	0	0	1	1	1	1	6	Good
Saez de Villareal et al. [67]	1	1	0	1	0	0	0	1	1	1	1	6	Good
Drouzas et al. [68]	1	1	0	1	0	0	0	0	1	1	1	5	Fair
Drury et al. [69]	1	1	0	1	0	0	0	1	1	1	1	6	Good
Ferrete et al. [70]	1	1	0	1	0	0	0	1	1	1	1	6	Good
Franco-Marquez et al. [34]	1	0	0	0	0	0	0	0	1	1	1	3	Poor
Hammami et al. [71]	1	1	0	1	0	0	0	1	1	1	1	6	Good
Hammami et al. [72]	1	1	1	1	0	1	0	1	1	1	1	8	Good
Hammami et al. [73]	1	1	0	1	0	0	0	1	1	1	1	6	Good
Hoshikawa et al. [74]	1	0	0	1	0	0	0	1	1	1	1	5	Fair
Keiner et al. [75]	1	0	0	1	0	0	0	1	1	1	1	5	Fair
Keiner et al. [28]	1	0	0	1	0	0	0	1	1	1	1	5	Fair
Keiner et al. [76]	1	0	0	1	0	0	0	1	1	1	1	5	Fair
Makhlouf et al. [77]	1	1	0	1	0	0	0	1	1	1	1	6	Good
Marques et al. [78]	1	1	0	1	0	0	0	1	1	1	1	6	Good
Michailidis et al. [29]	1	1	0	1	0	0	0	0	1	1	1	5	Fair
Negra et al. [79]	1	1	0	1	0	0	0	0	1	1	1	5	Fair
Negra et al. [80]	1	1	0	1	0	0	0	1	1	1	1	6	Good
Negra et al. [81]	1	1	0	1	0	0	0	1	1	1	1	6	Good
Padron-Cabo et al. [82]	1	1	0	1	0	0	0	0	1	1	1	5	Fair
Pena-Gonzalez et al. [83]	1	1	0	1	0	0	0	1	1	1	1	6	Good
Ramirez-Campillo et al. [87]	1	1	1	1	0	0	0	1	1	1	1	7	Good
Raya-Gonzalez et al. [84]	1	1	1	1	0	0	0	1	1	1	1	7	Good
Rodriguez-Rosell et al. [85]	1	1	0	1	0	0	0	1	1	1	1	6	Good
Szymanek-Pilarczyk [86]	1	0	0	1	0	0	0	1	1	1	1	5	Fair
Wong et al. [35]	1	0	0	1	0	0	0	0	1	1	1	4	Fair

^a^From a possible maximal score of 10 (item 1 is not included in the total)

Across the included studies, there was a total sample size of *n* = 1396 high-level, highly trained youth male soccer players, of which *n* = 327 participated in strength training, *n* = 217 in plyometric training, *n* = 308 in combined training and *n* = 534 acted as active controls. Descriptive characteristics of participants and the training programme design for included studies are provided in Table 2. Training studies lasted for 6–104 weeks, with 79% of studies having a duration of ≤ 12 weeks. Most studies (82%) included a training frequency of twice per week, although the range in frequency was one to four times per week. Where reported, the intensity of plyometric training was always maximal or near-maximal (e.g. maximal effort), whereas strength and combined training was completed at intensities that ranged from 40% one-repetition maximum to maximal efforts. Details on the repetition range, number of sets, recovery between sets and recovery between sessions are provided in Table 2, with more detailed information on the exercises included in each programme provided in the ESM.

**Table 2:** Participant characteristics and training programme design of studies examining the effects of strength, plyometric or combined training on the physical fitness of high-level, highly trained young soccer players

Study	Sample size (*n*)	Age (years)	Academy/competition level	Training (hours/week)	Frequency (per week)	Duration (weeks)	Intensity	Repetitions (*n*)	No. of sets (*n*)	Rest between sets (s)	Min. recovery between sessions (h)
Abade et al. [88]	STG1 (vertical): 8 STG2 (horizontal): 8 CON: 8	16.56 ± 0.56	National league	7 h 20 min	1	20	NR	4–10	3	90	NR
Chelly et al. [26]	STG: 11 CON: 11	STG: 17 ± 0.3 CON: 17 ± 0.5	National junior soccer championship	8 h	2	8	70–90% 1RM	2–7	NR	NR	48
Drury et al. [69]	STG: 8 CON:11	STG: 11 ± 0.9 CON:10.9 ± 0.8	NR	6 h	1–2	6	NR	5–8	2–3	60–90	48
Hoshikawa et al. [74]	STG: 16 CON: 12	12–13.1	NR	10 h	4	24	NR	30 s, 10 reps	1–2	NR	NR
Keiner et al. [76]	U17: STG: 30 CON: 21 U15: STG: 18 CON: 17	U17, U15	First and second division clubs	NR	2	104	NR	4–10	5	180–300	NR
Negra et al. [80]	STG: 13 CON: 11	STG: 12.8 ± 0.25 CON: 12.74 ± 0.26	Regional soccer team	NR	2	12	40–60% 1RM	8–15	4–6	120	72
Pena-Gonzalez et al. [83]	STG: Pre-PHV: 43 Mid-PHV: 36 Post-PHV: 31 CON: 20	STG: Pre-PHV: 12.8 ± 0.4 Mid-PHV: 13.8 ± 0.6 Post-PHV: 14.6 ± 0.5 CON: 13.2 ± 1.1	Spanish regular league competition	5 h 10 min	2	8	Maximal	NR	NR	1:1	NR
Raya-Gonzalez et al. [84]	STG: 10 CON: 10	U16	Elite Spanish soccer club	5 h	1	10	As fast as possible	8–10	2–4	180	168
Wong et al. [35]	STG: 28 CON: 23	STG: 13.5 ± 0.7 CON: 13.2 ± 0.6	Highest level in Hong Kong	4 h	2	12	NR	5–15	3	< 30	NR
Hammami et al. [71]	STG: 14 PTG: 14 CON: 12	STG: 16.1 ± 0.5 PTG: 15.7 ± 0.2 CON: 15.8 ± 0.2	Elite level championship	6–7 h 30 min	2	8	70–90% 1RM Maximal	3–8 7–10	3–5 4–10	NR NR	48 NR
Keiner et al. [75]	STG1: 14	17.45 ± 0.52	Highest German division national or state league	NR	2	40	Medium to strong load improvement maximal	10 10 6–8	2–5 3–5 6–8	120 180 300	48
	STG2: 11										
	CTG: 11 CON: 12										
Negra et al. [81]	STG: 12 PTG: 11 CON: 11	STG: 12.8 ± 0.3 PTG: 12.7 ± 0.3 CON: 12.8 ± 0.3	Regional soccer team	NR	2	12	40–60% 1RM Maximal	10–15 112–280 gcs	4–6 NR	NR 90	72
Chtara et al. [66]	PTG: 10 CON: 10	13.6 ± 0.3	Tunisian first league	6 h	2	6	Sub-maximal to maximal	8–15	2–3	90	48–72
Drouzas et al. [68]	PTG1: 23 PTG2: 23 CON: 22	PTG1: 9.9 ± 1.8 PTG2: 10 ± 0.5 CON: 10.2 ± 1.6	Greek Super league club	3 h	2	10	Maximal	6–10	3–5	1:5–1:10	NR
Hammami et al. [72]	PTG1: 12 PTG2: 14 CON: 12	PTG1: 16.2 ± 0.2 PTG2: 16.3 ± 0.4 CON: 16.4 ± 0.2	International tournaments	6–8 h	2	10	Maximal	2–6	NR	30	48
Hammami et al. [73]	PTG: 15 CON: 13	PTG: 15.7 ± 0.2 CON: 15.8 ± 0.2	National championship		2	8	Maximal	7–10	4–10	NR	NR
Michailidis et al. [29]	PTG: 24 CON: 21	PTG: 10.7 ± 0.7 CON: 10.6 ± 0.5	NR	3 h 30 min to 6 h	2	12	Low to moderate	5–10	2–4	90–180	72
Negra et al. [79]	PTG: 13 CON: 11	PTG: 12.7 ± 0.2 CON: 12.7 ± 0.2	NR	6 h 30 min to 7 h 30 min	2	8	Maximal	10–15	5–8	90	72
Padron-Cabo et al. [82]	PTG: 10 CON: 10	PTG: 12.6 ± 0.7 CON: 12.4 ± 0.6	Competitive season	8–10 h training + 1 match	2	6	Maximal	6–14	1–4	120	48
Ramirez-Campillo et al. [87]	PTG1: 12 PTG2: 14 CON: 12	PTG1: 16.9 ± 0.7 PTG2: 17.1 ± 0.3 CON: 17.1 ± 0.5	National level	1 h 30 min to 2 h	2	7	NR	4–16	1	30–60	72–96
Szymanek-Pilarczyk [86]	PTG: 24 CON: 21	PTG: 16–18 CON: 16–18	Polish first division	NR	NR	8	NR	6–16	2–4	NR	NR
Aloui et al. [89]	CTG: 17 CON: 17	CTG: 14.6 ± 0.5 CON: 14.6 ± 0.4	National first division	7 h 30 min	2	8	NR	72–144 gcs	3–6	90	NR
Aloui et al. [92]	CTG: 17 CON: 17	CTG: 16.5 ± 0.5 CON: 16.7 ± 0.5	National first division	7 h 30 min	2	8	Maximal height and distance	72–144 gcs	3–6	NR	NR
Aloui et al. [91]	CTG: 18 CON: 16	CTG: 16.6 ± 0.5 CON: 16.6 ± 0.5	Tunisia national team first division	7 h 30 min	2	8	NR	72–144 gcs	3–6	NR	NR
Aloui et al. [90]	CTG: 17 CON: 16	CTG: 16.6 ± 0.5 CON: 16.6 ± 0.5	National first division	7 h 30 min	2	8	NR	72–144 gcs	3–6	NR	NR
Maio Alves et al. [64]	CTG1: 9 CTG2: 8 CON: 6	17.4 ± 0.6	Portuguese elite championship	NR	1 2	6	80–90% 1RM	1–6	NR	NR	NR
Boraczynski et al. [65]	CTG1: 22 CTG2: 24 CON: 21	CTG1: 11.2 ± 0.4 CTG2: 11.1 ± 0.2 CON: 11 ± 0.3	NR	4 h 30 min	3	24	85–95% HRmax 75–85% HRmax	Moderate to high	2	30	48
Saez de Villareal et al. [67]	CTG: 13 CON: 13	CTG: 15.33 ± 0.34 CON: 14.90 ± 0.17	Spanish first division	NR	2	9	Maximal	6–10	2–4	60	72
Ferrete et al. [70]	CTG: 11 CON: 13	CTG: 9.3 ± 0.3 CON: 8.3 ± 0.3	Spanish first division	NR	2	26	NR	4–10	2–4	60	NR
Franco-Marquez et al. [34]	CTG: 20 CON: 18	CTG: 14.7 ± 0.5 CON: 14.7 ± 0.5	National first division	9 h 10 min	2	6	Maximal	2–6	5–10	180	> 24
Keiner et al. [28]	CTG1: 19 CTG2: 19 CON: 16	CTG1: 11.5 ± 0.5 CTG2: 9.5 ± 0.5	Second/third division	> 5 h training	2	104	NR	6–15	3–5	120	NR
Makhlouf et al. [77]	CTG1: 21 CTG2: 20 CON: 16	CTG1: 11.1 ± 0.8 CTG2: 11.3 ± 0.9 CON: 11 ± 0.8	U12 National championship	NR	2	8	Maximal	8–15	1–3	NR	NR
Marques et al. [78]	CTG: 26 CON: 26	13.4 ± 1.4	National level	NR	2	6	Maximal	4–25	2–6	NR	NR
Rodriguez-Rosell et al. [85]	CTG: 15 CON: 15	CTG: 12.7 ± 0.5 CON: 12.8 ± 0.5	NR	9 h 10 min training + 1 match	2	6	45–58% 1RM	4–20	2–5	180	72

*1RM* one-repetitionmaximum, *CON* control, *CTG* combined training group, *gcs* ground contacts, *h* hours, *HR* heart rate, *max* maximum, *mins* minutes, *PE* physical education, *PHV* peak height velocity, *PTG* plyometric training group, *NR* not reported, *reps* repetitions, *RM* repetition maximum, *s* seconds, *STG* strength training group, *U* under

First division relates to the top national division (e.g. academy in a La Liga club)

### Meta-Analysis

**Fig. 2:**
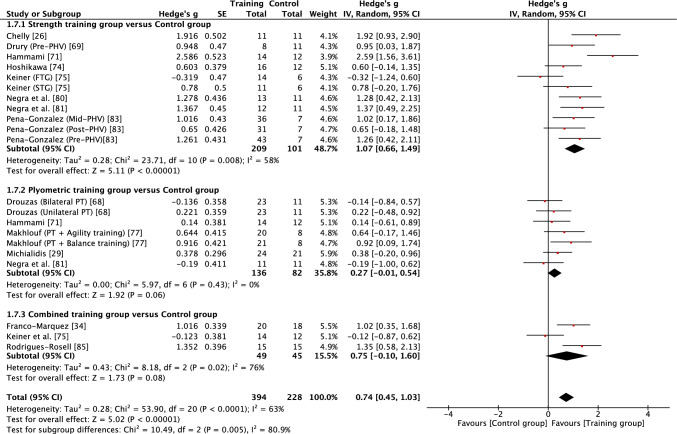
Forest plot showing the effects of strength (top), plyometric (middle) and combined (bottom) training on lower body strength in high-level, highly trained youth male soccer players. *FTG* functional training group, *PHV* peak height velocity, *PT* plyometric training, *STG* strength training group

**Fig. 3:**
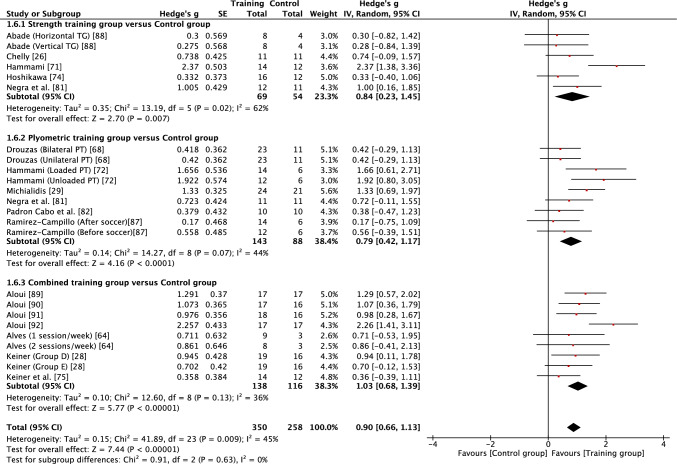
Forest plot showing the effects of strength (top), plyometric (middle) and combined (bottom) training on squat jump performance in high-level, highly trained youth male soccer players. *PT* plyometric training, *TG* training group

**Fig. 4:**
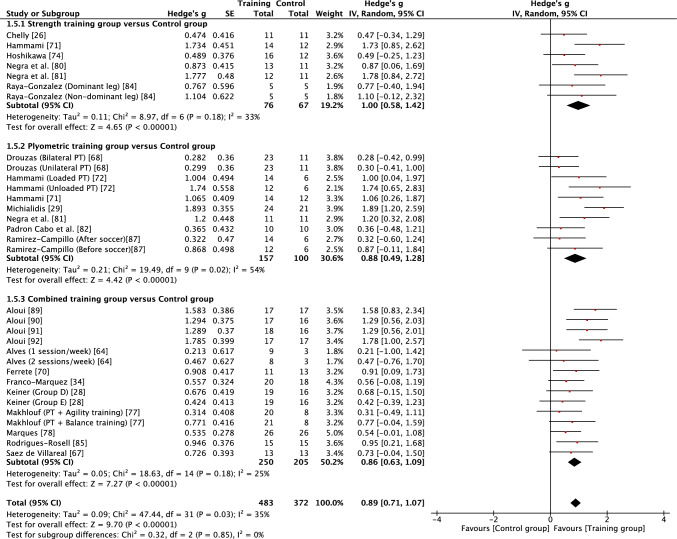
Forest plot showing the effects of strength (top), plyometric (middle) and combined (bottom) training on countermovement jump performance in high-level, highly trained youth male soccer players. *PT* plyometric training

**Fig. 5:**
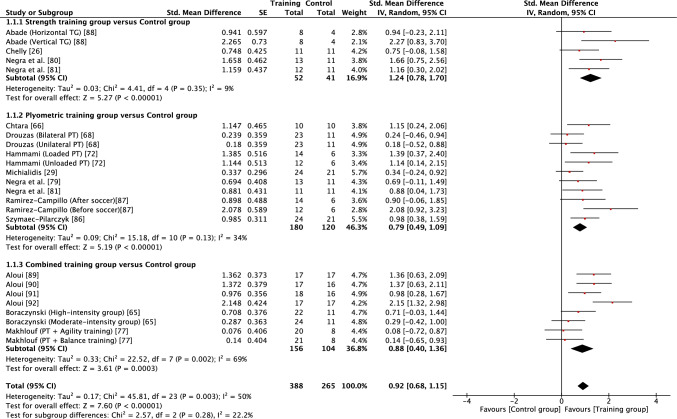
Forest plot showing the effects of strength (top), plyometric (middle) and combined (bottom) training on horizontal power in high-level, highly trained youth male soccer players. *PT* plyometric training, *TG* training group

**Fig. 6:**
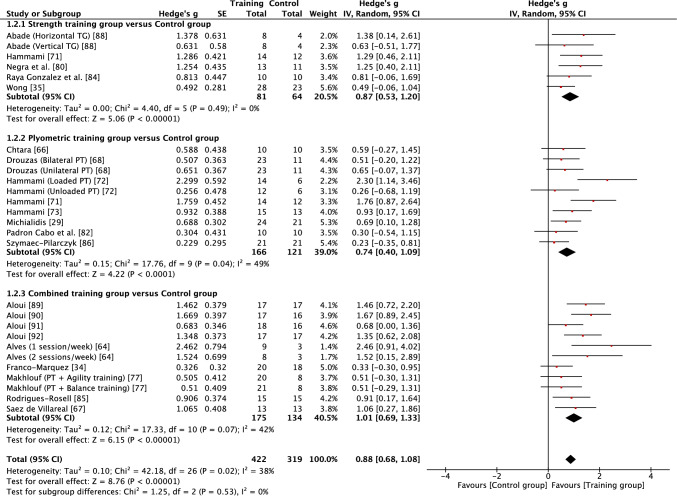
Forest plot showing the effects of strength (top), plyometric (middle) and combined (bottom) training on acceleration in high-level, highly trained youth male soccer players. *PT* plyometric training, *TG* training group

**Fig. 7:**
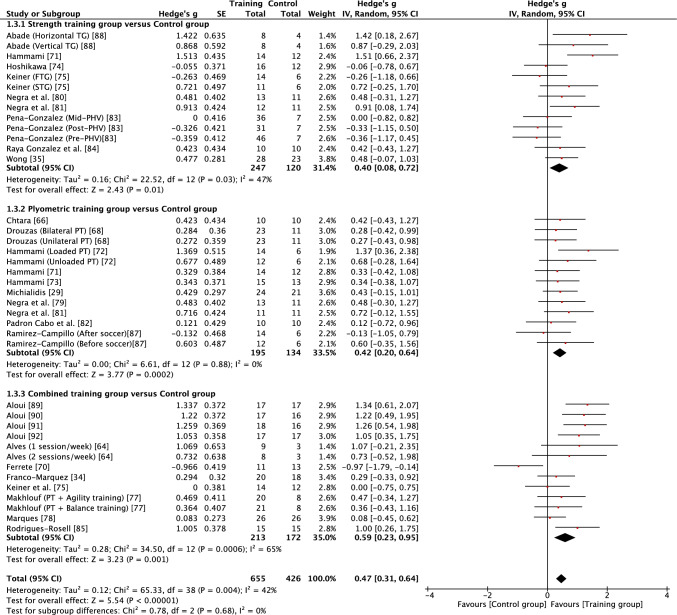
Forest plot showing the effects of strength (top), plyometric (middle) and combined (bottom) training on sprint speed in high-level, highly trained youth male soccer players. *FTG* functional training group, *PHV* peak height velocity, *PT* plyometric training, *STG* strength training group, *TG* training group

**Fig. 8:**
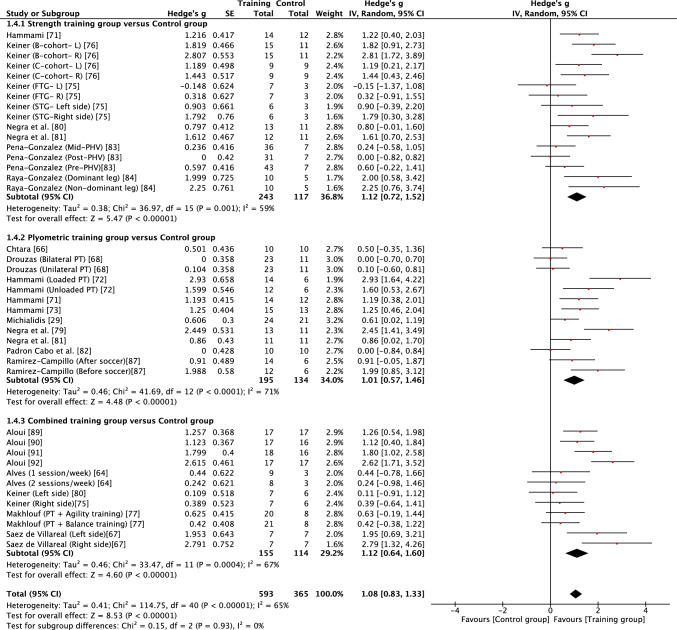
Forest plot showing the effects of strength (top), plyometric (middle) and combined (bottom) training on change of direction speed in high-level, highly trained youth male soccer players. *FTG* functional training group,* L* left, *PHV* peak height velocity, *PT* plyometric training, *R* right, *STG* strength training group, *TG* training group

Forest plots showing the effects of the different modes of training on strength, squat jump, countermovement jump, horizontal power, acceleration, speed and CODS are shown in Figs. 2, 3, 4, 5, 6, 7, 8, respectively. Summary findings of the meta-analysis across all fitness outcomes, both in terms of intervention effects and measures of heterogeneity, are provided in Table 3.

**Table 3:** Summary outcomes of the meta-analyses examining the effects of strength, plyometric and combined training on measures of physical fitness in high-level, highly trained young soccer players

Outcome measure	Training intervention	No of studies (*n*)	No. of groups (EG vs CON)	Total sample (EG vs CON)	Intervention effects			Measure of heterogeneity		
					*p* value	Effect size (*g*)	Effect	*I*^2^ value	*χ*^2^ (*p* value)	Prediction interval
Lower body strength	STG vs CON	7	11 vs 7	209 vs 101	< 0.001	1.07 (0.66–1.49)	Moderate	58%	0.0080	− 0.22 to 2.36
	PTG vs CON	5	7 vs 5	136 vs 82	0.060	0.27 (− 0.01 to 0.54)	Small	0%	0.430	− 0.08 to 0.62
	CTG vs CON	3	3 vs 3	49 vs 45	0.080	0.75 (− 0.10 to 1.60)	Moderate	76%	0.020	− 9.24 to 10.74
	***Overall***	***15***	***21 vs 15***	***394 vs 228***	** < *0.001***	***0.74 (0.45***–***1.03)***	***Moderate***	***63%***	** < *0.001***	− ***0.41 to 1.89***
Squat jump	STG vs CON	5	6 vs 5	69 vs 54	0.007	0.84 (0.23–1.45)	Moderate	62%	0.020	− 1.02 to 2.70
	PTG vs CON	6	9 vs 6	143 vs 88	< 0.001	0.79 (0.42–1.17)	Moderate	44%	0.070	− 0.21 to 1.79
	CTG vs CON	7	9 vs 7	138 vs 116	< 0.001	1.03 (0.68–1.39)	Moderate	36%	0.130	0.17–1.89
	***Overall***	***18***	***24 vs 18***	***350 vs 258***	** < *0.001***	***0.90 (0.66***–***1.13)***	***Moderate***	***45%***	***0.009***	***0.06–1.74***
Counter movement jump	STG vs CON	6	7 vs 5	76 vs 67	< 0.001	1.00 (0.58–1.42)	Moderate	33%	0.180	− 0.02 to 2.02
	PTG v CON	7	10 vs 7	157 vs 100	< 0.001	0.88 (0.49–1.28)	Moderate	54%	0.020	− 0.28 to 2.04
	CTG vs CON	12	15 vs 12	250 vs 205	< 0.001	0.86 (0.63–1.09)	Moderate	25%	0.180	0.31–1.41
	***Overall***	***25***	***32 vs 24***	***483 vs 372***	** < *0.001***	***0.89 (0.71***–***1.07)***	***Moderate***	***35%***	***0.030***	***0.25***–***1.53***
Horizontal power	STG vs CON	4	5 vs 4	52 vs 41	< 0.001	1.24 (0.78–1.70)	Large	9%	0.350	0.31–2.17
	PTG vs CON	8	11 vs 8	180 vs 120	< 0.001	0.79 (0.49–1.09)	Moderate	34%	0.130	0.03–1.55
	CTG vs CON	6	8 vs 6	156 vs 104	< 0.001	0.88 (0.40–1.36)	Moderate	69%	0.002	− 0.65 to 2.41
	***Overall***	***18***	***24 vs 18***	***388 vs 265***	** < *0.001***	***0.92 (0.68***–***1.15)***	***Moderate***	***50%***	***0.003***	***0.03***–***1.81***
Acceleration (0–10 m)	STG vs CON	5	6 vs 5	81 vs 64	< 0.001	0.87 (0.53–1.20)	Moderate	0%	0.49	0.40–1.34
	PTG vs CON	8	10 vs 8	166 vs 121	< 0.001	0.74 (0.40–1.09)	Moderate	49%	0.04	− 0.24 to 1.72
	CTG vs CON	9	11 vs 9	175 vs 134	< 0.001	1.01 (0.69–1.33)	Moderate	42%	0.07	0.14–1.88
	***Overall***	***22***	***27 vs 22***	***422 vs 319***	** < *0.001***	***0.88 (0.68***–***1.08)***	***Moderate***	***38%***	***0.02***	***0.20***–***1.56***
Change of direction speed	STG vs CON	7	16 vs 7	243 vs 117	< 0.01	1.12 (0.72–1.52)	Moderate	59%	0.001	− 0.27 to .51
	PTG vs CON	10	13 vs 10	195 vs 134	< 0.01	1.01 (0.57–1.46)	Moderate	71%	< 0.001	− 0.57 to 2.59
	CTG vs CON	8	12 vs 8	155 vs 114	< 0.05	1.12 (0.64–1.60)	Moderate	67%	< 0.001	− 0.49 to 2.73
	***Overall***	***25***	***41 vs 25***	***593 vs 365***	** < *0.01***	***1.08 (0.83***–***1.33)***	***Moderate***	***65%***	** < *0.001***	− ***0.25 to 2.4***
Speed (15–40 m)	STG vs CON	9	13 vs 9	247 vs 120	0.010	0.40 (0.08–0.72)	Small	47%	0.030	− 0.55 to 1.35
	PTG vs CON	10	13 vs 10	195 vs 134	< 0.001	0.42 (0.20–0.64)	Small	0%	0.88	0.17–0.67
	CTG vs CON	11	13 vs 11	213 vs 172	0.001	0.59 (0.23–0.95)	Small	65%	< 0.001	− 0.64 to 1.82
	***Overall***	***30***	***39 vs 30***	***655 v 426***	** < *0.001***	***0.47 (0.31***–***0.64)***	***Small***	***42%***	***0.004***	− ***0.25 to 1.19***

*CI* confidence interval, *CON* control group, *CTG* combined training group, *EG* experimental group, *PTG* plyometric training group, *STG* strength training group

When considering the effects of each type of intervention (strength, plyometric or combined training) or overall effects (all interventions combined), results revealed that in most cases training effects were significant and moderate in size (see Table 3). This included all improvements being reported as moderate for all forms of training with regard to squat jump, countermovement jump, linear acceleration and CODS, and overall effects as moderate for all outcomes other than linear speed. While all forms of training resulted in significant improvements in linear speed (all* p* < 0.05), all effects were small in magnitude (*g* = 0.40–0.59). As a result of the general consistency of effects across training, the type of training was found to have no significant effect on all fitness outcomes (*p* > 0.05) except lower body strength. For lower body strength, there was a significant effect (*p* < 0.05) of training mode, reflecting the moderate positive effects of strength and combined training (*g* = 1.07 and 0.75, respectively) and only small positive effects of plyometric training (*g* = 0.27). While the mode of training did not have a significant effect on horizonal jump performance, it is noteworthy that strength training resulted in large improvements (*g* = 1.24), compared with only moderate improvements from plyometric and combined training (*g* = 0.79 and 0.88, respectively). When all fitness outcomes were combined, there was a tendency for plyometric training (*g* = 0.71, 95% CI 0.57–0.84) to result in a lower positive effect than strength (*g* = 0.91, 95% CI 0.74–1.08) and combined (g = 0.89, 95% CI 0.74–1.03) training, but the effect did not reach significance (*p* = 0.10).

Measures of heterogeneity are shown in Table 3. Usually (21/28), the *χ*^2^ test was significant (*p* < 0.05), including for all overall effects. In four cases where the *χ*^2^ statistic was non-significant (*p* > 0.05), the *I*^2^ statistic also revealed low levels of heterogeneity. However, in most cases, *I*^2^ results suggested moderate levels of heterogeneity, with only a single case of a high level of heterogeneity. Heterogeneity was reflected in some level of uncertainty in prediction intervals, with intervals often crossing zero. However, prediction intervals for overall training effects were entirely positive for squat jump, countermovement jump, horizontal jump and linear acceleration, reflecting some positive prediction intervals for individual modes of training within those fitness outcomes (see Table 3). Although the effect of plyometric training on speed was small (*g* = 0.42), the prediction interval was entirely positive (0.17–0.67).

## Discussion

The aim of this systematic review and meta-analysis was to examine whether highly trained male youth soccer players could improve strength, power and speed characteristics in response to strength, plyometric and combined training, compared with standard soccer training. The major findings show that this unique population made significant and meaningful gains across all forms of training and fitness outcomes following what were typically short duration (≤ 12 weeks) interventions. Nearly all training outcomes across strength, squat and countermovement jump, horizontal power, acceleration and CODS led to moderate improvements in fitness. Lower body strength was the only outcome to be significantly influenced by the mode of training, with moderate gains following strength and combined training compared with only small gains with plyometric training. Improvements to speed were more blunted and while significant, they were only small in magnitude following all forms of training. Some caution should be taken with the generalisability of findings, given the potential for publication bias in some findings and moderate levels of heterogeneity. Nevertheless, when considering the overall outcomes, prediction intervals suggest future strength, plyometric and combined training in high-level, highly trained male youth soccer players is likely to lead to positive improvements in squat and countermovement jump performance, horizontal power and acceleration, with overall effects moderate in magnitude. While overall effects demonstrated moderate effects of training for strength and CODS, and small effects for speed, some caution should be taken when considering the transference of these effects to future populations, given the wider prediction intervals.

The magnitudes of effect for improvements in lower body strength were approximately three to four times greater using combined training or strength training when compared with plyometric training, suggesting an effect of training specificity. Our finding is similar to a previous meta-analysis that combined trained and untrained youth. Behm et al. [39] reported that following strength training, adolescents experienced a moderate increase in strength (*g* = 0.88), but only a trivial improvement in strength following power training (*g* = 0.16). While the population in the current study were highly trained, their normal soccer training may have previously exposed them to very little strength training, meaning that baseline strength was relatively low. When describing the training practices of academy soccer players, it has been noted that only 7–14% of their weekly training load is attributed to work in a gym [20]. Although strength training experience was not typically reported in the studies included in this review, some studies did report that players had no prior strength training experience [26, 69]. In the present review, combined training was also found to positively improve lower body strength, albeit with a lower effect size compared with strength training (*g* = 0.75 vs 1.07). The positive response to combined training may be partly explained by the fact that all studies included some element of strength training as part of the combined training programme [34, 75, 85]. Findings from this review suggest that strength training provides a specific response in terms of improving strength in high-level, highly trained male youth soccer players, and positive responses may be partly related to a low strength training history in this population. Strength and strength training have been suggested to be important in youth soccer because of associations with both physical performance [24–27, 93, 94] and injury prevention [95, 96]. Given the importance of strength and the unique stimulus provided by strength training, including within combined training, it would appear desirable to include this mode of training in the development of young, high-level, highly trained male soccer players.

While all forms of training led to significant and moderate improvements in acceleration, training only transferred to small gains in speed. This may be because of the differing mechanical determinants of acceleration and speed in highly trained youth soccer players [97], and the ability of different types of training to target adaptations in these determinants. Plyometric training is the most popular type of training used to increase maximal speed by strength and conditioning coaches working in elite soccer, while strength training is also popular [98]. However, these types of training may lack specificity to maximal speed production. In boys aged 11–16 years, maximal sprint speed has been shown to be determined by relative stiffness, requiring high levels of force to be generated during very brief ground contact periods (~ 140 ms) while resisting vertical displacement of the centre of mass during ground contact, all while trying to propel the body forward [99]. Although maximal speed and plyometric exercise may both rely on the stretch–shortening cycle by using pre-activation and stretch reflexes to generate high levels of speed and power [100], they may not always be well matched. In the studies included in the current meta-analysis of speed, plyometric training often included exercises that were vertical or lateral in direction, exercises involving large joint range of movement and centre of mass displacement (e.g. countermovement jump, drop jump from a large height), and exercises that would likely involve use of a slow-stretch shortening cycle (e.g. ground contact time > 250 ms) [66, 68, 71–73, 79, 87]. Consequently, the mechanical and neuromuscular demands imposed by the plyometric training may have had greater training transfer to acceleration and CODS, rather than maximal sprint speed. Strength, plyometric and combined training can result in small gains in speed in high-level, highly trained youth soccer players; however, training for speed may need to be more specific to achieve greater gains. Training specificity for maximal speed development could be further improved by focusing on exercises that target a fast stretch–shortening cycle, requiring high levels of stiffness and rapid horizontal force production. However, more research is needed to understand how highly trained male youth soccer players respond to resistance training that is more targeted towards improving maximal speed.

The squat jump, countermovement jump, horizontal power, acceleration and CODS all appeared to be trainable to a similar extent, with consistent moderate gains for all forms of training and a large effect of strength training on horizontal power. Highly trained youth soccer players are likely to spend a considerable amount of time in match play or playing small-sided games during training [101], with young players frequently sprinting [102] and likely often jumping during match play and with small-sided games imposing high acceleration and deceleration demands on young players [103]. Despite highly trained male youth soccer players habitually engaging in brief explosive actions as part of their normal training and competition, strength, plyometric and combined training were all effective at further improving jump, acceleration and change of direction performance. It has been stated that jumping, acceleration and change of direction ability are all independent qualities that should be trained separately in adult soccer players [104]. Of note, the findings from this review suggest some common transference of training adaptations to multiple fitness outcomes, which may not be surprising given known associations that have been reported between strength, jump, sprint and change of direction qualities in youth soccer players [24–27, 93, 94]. Based on prediction intervals of overall effects, the findings of the current review also show that practitioners can have reasonable confidence that future training will lead to positive outcomes in squat and countermovement jump, horizontal power and acceleration in high-level, highly trained youth male soccer players.

It is likely those working to support the physical development of youth soccer players will want to simultaneously target the development of multiple fitness outcomes. Consequently, identifying modes of training with multiple fitness benefits is desirable. All modes of training considered in the current review resulted in significant positive benefits in all measures of strength, power and speed. However, when fitness outcomes were combined, the overall effect of plyometric training was lower compared with strength and combined training (*g* = 0.72 vs 0.91 vs 0.89, respectively), albeit this difference was not significant (*p* = 0.10). The difference in the magnitude of response across training modes may reflect the fact that strength and combined training provide a more novel training stimulus in this population, while plyometric training may somewhat repeat the routine use of the stretch–shortening cycle movement that is completed in soccer. This finding may support an approach that prioritises the use of strength or combined training with highly trained youth soccer players, particularly when considering the greater effects of strength training compared with plyometric training on strength (g = 1.07 vs 0.27) and horizontal power (g = 1.24 vs 0.79). However, long-term athletic development programmes should expose young athletes to a variety of training stimuli [21, 105] and plyometric training still provided clear benefits in the present review. It is also worth noting that most studies included in this review used short-duration interventions (≤ 12 weeks), and player development should consider a long-term approach and the inclusion and periodisation of a number of complementary training modes.

## Limitations

To the authors knowledge, this is the first review and meta-analysis to specifically focus on a highly trained sample of youth male athletes from a single sport and it is acknowledged that defining what constitutes “high level” or “highly trained” comes with some limitations. While the weekly training hours reported for some studies appear low (e.g. as low as 1.5–2 h per week [87]), participants in those studies identified that participants were competing at a high level (e.g. international, national leagues) and it may be that participants were accumulating more training hours outside of representative teams. Similarly, weekly training volume was often not reported, although in those instances the competitive standard was reported and identified players as high level. A definition for “highly trained” was based on training hours reported by academies from top clubs across European football [49]. While it may be possible that some lower level players could be attaining these high levels of training volume, the authors consider this to be unlikely. More importantly, any players meeting the criteria to be classified as highly trained will have been exposed to a large volume of systematic football training, reflecting the purpose of this review to explore the ability of young players who have accumulated substantial soccer-training history to respond to different forms of resistance training. By limiting the review to high-level, highly trained male youth soccer players, the overall pool of studies and total sample size that could be included was restricted, which in turn may have contributed to some publication bias across certain outcomes, with a slight over-representation of small studies with larger effects (and the under-representation of small studies with limited effects). Nevertheless, the total sample size was similar to notable meta-analyses that have investigated the effects of resistance training in youth athletes across sports [41], and in the general population of children and adolescents [38]. Moreover, the focus on high-level male youth soccer players reduced heterogeneity arising from the incorporation of more divergent groups. The review excluded female players, partly owing to a greater difficulty in establishing training volumes associated with a high level in this population, partly because of a lack of available research in this population, and partly because of the differing maturation processes in boys and girls and the potential confounding effects on training responses.

The quality was good for most studies, but only fair for some studies, and was poor for one study. Nearly all studies failed to blind participants, coaches or assessors to the intervention condition, and this will likely be the case in future research. Future research should implement randomisation procedures, ensuring a similar baseline level of key dependent variables. Heterogeneity was also present to some degree in most outcomes. To account for the effects of heterogeneity on meta-analytic outcomes, prediction intervals were calculated to give a measure of certainty in future training effects. Given the mode of training did not significantly affect most outcomes, it seems appropriate to consider the prediction interval from the overall effects for each fitness outcome. Taking this approach demonstrates that despite the presence of heterogeneity, reasonable confidence can be taken that future training effects would likely lead to positive outcomes, particularly for jump performance, horizontal power and acceleration. An analysis of the moderating effects of programme design (e.g. intensity, frequency and duration of interventions) and participant characteristics (e.g. maturity, baseline fitness) could have provided an interesting perspective. Unfortunately, the relatively small sample sizes, difficulty in equating programme variables, such as intensity, across different forms of training, and the inconsistency in reporting of factors such as maturity status, precluded any meaningful analysis of training moderators. Data were pooled within each fitness quality across a number of different tests and variables, which may limit some of the findings. For instance, COD responses to training may be very specific to both the type of training and the type of COD movement and test. More data would be required to fully examine such factors.

## Conclusions

The purpose of this systematic review and meta-analysis was to examine the specific effects of strength, plyometric and combined training on the strength, power and speed characteristics of high-level, highly trained male youth soccer players. Overall, the findings demonstrated that high-level, highly trained male youth soccer players can make positive improvements across strength, power and speed outcomes following exposure to strength, plyometric and combined training. These gains were typically moderate in magnitude following exposure to short duration (≤ 12 weeks) training interventions. Mode of training had a limited influence on the magnitude of training effects, although strength gains did differ with the type of training. The findings of this review support the inclusion of strength training in the physical development of highly trained male youth soccer players, given that this mode of training provided a specific stimulus to increase strength and resulted in greater (albeit non-significant) gains in horizontal power. Despite the presence of heterogeneity, results suggest with reasonable confidence that future training effects in this unique population would likely be positive, particularly when considering squat and countermovement jump, horizontal power and acceleration. While strength, plyometric and combined training can significantly improve the speed of high-level, highly trained male youth soccer players, gains are likely to be only small in magnitude. Collectively, the findings indicated that the included training programmes more closely reflected and overloaded the mechanical and neuromuscular demands of jumping, bounding, accelerating and decelerating, but were less specific to the horizontal force demands of maximal sprint speed. Future research should clearly report the training history, participant characteristics (e.g. maturity) and programme design, so the effect of these moderators on training can be fully examined.

### Supplementary Information

Below is the link to the electronic supplementary material.

Supplementary file1 (DOCX 208 KB)

## Declarations

### Availability of data and material

The datasets generated and/or analysed during the current study are available from the corresponding author on reasonable request.
