# Supplementary material for: The Effects of Strength, Plyometric and Combined Training on Strength, Power and Speed Characteristics in High-Level, Highly Trained Male Youth Soccer Players: A Systematic Review and Meta-Analysis
Source: Sports Med. 2023 Oct 28;54(3):623–43. doi: 10.1007/s40279-023-01944-8 (PMC10978689; doi:10.1007/s40279-023-01944-8)
Supplement: Supplementary file 1 — Supplementary file1 (DOCX 208 KB) [file 40279_2023_1944_MOESM1_ESM.docx]

**Title: The effects of strength, plyometric and combined training on strength, power and speed characteristics in high-level, highly-trained male youth soccer players: a systematic review and meta-analysis**

**Journal Name: Sports Medicine**

**Authors:** Jon L. Oliver^1,2^, Akhilesh Kumar Ramachandran^1^, Utkarsh Singh^3^, Rodrigo Ramirez-Campillo^4^, Rhodri S. Lloyd^1,2^

**Affiliations:** ^1^Youth Physical Development Centre, Cardiff School of Sport and Health Sciences, Cardiff Metropolitan

University, Cardiff CF23 6XD, United Kingdom.

^2^Sport Performance Research Institute New Zealand (SPRINZ), Auckland University of Technology, New Zealand

^3^Sports and Exercise Science, College of Healthcare Sciences, James Cook University, Townsville, Australia QLD4811.

^4^Exercise and Rehabilitation Sciences Institute. School of Physical Therapy. Faculty of Rehabilitation Sciences. Universidad Andres Bello. Santiago 7591538, Chile.

**Corresponding author**

Jon Oliver: [joliver@cardiffmet.ac.uk](mailto:joliver@cardiffmet.ac.uk)

ORCID iD: 0000-0001-7425-3148

**Supplementary Material 1:** Database Search Terms

1. **PubMed**

(“youth” [Title/Abstract] OR “Adolescent*” [Title/Abstract] OR “Child*” [Title/Abstract] OR “Young” [Title/Abstract] OR “pubert*” [Title/Abstract]) AND (“soccer” [Title/Abstract] OR “football” [Title/Abstract]) AND (“intervention*” [Title/Abstract] OR “training” [Title/Abstract]) AND (“strength” [Title/Abstract] OR “plyometric” [Title/Abstract] OR “combine*” [Title/Abstract] OR “Jump” [Title/Abstract] OR “Explosive” [Title/Abstract] OR “ballistic” [Title/Abstract])) = **611 results**

1. **Scopus**

(TITLE-ABS-KEY (“youth” OR “Adolescent*” OR “Child*” OR “Young” OR “pubert*”)) AND (TITLE-ABS-KEY (“soccer” OR “football”)) AND (TITLE-ABS-KEY (“intervention*” OR “training”)) AND (TITLE-ABS-KEY ((“strength” OR “plyometric” OR “combine*” OR “Jump” OR “Explosive” OR “ballistic”)) = **2,023 results**

1. **SPORTDiscus**

(“youth” OR “Adolescent*” OR “Child*” OR “Young” OR “pubert*”) AND (“soccer” OR “football”) AND (“intervention*” OR “training”) AND (“strength” OR “plyometric” OR “combine*” OR “Jump” OR “Explosive” OR “ballistic”) = **1,063 results**

1. **CINAHL**

("youth" OR "Adolescent*" OR "Child*" OR "Young" OR "pubert*") AND (soccer OR football) AND (“intervention*” OR “training”) AND (“strength” OR “plyometric” OR “combine*” OR “Jump” OR “Explosive” OR “ballistic”) = **467 results**

1. **Web of Science**

Topic (“youth” OR “Adolescent*” OR “Child*” OR “Young” OR “pubert*”) AND Topic (“soccer” OR “football”) AND Topic (“intervention*” OR “training”) AND Topic (“strength” OR “plyometric” OR “combine*” OR “Jump” OR “Explosive” OR “ballistic”) = **1,300** **results**

**Supplementary file 2**

**Fig 9:** Funnel plots for assessing publication bias for the following outcomes: top-left: lower body strength; top-centre: squat jump; top-right: countermovement jump; middle-left: horizontal power; middle-centre: acceleration; middle-right: change of direction; bottom-left: speed


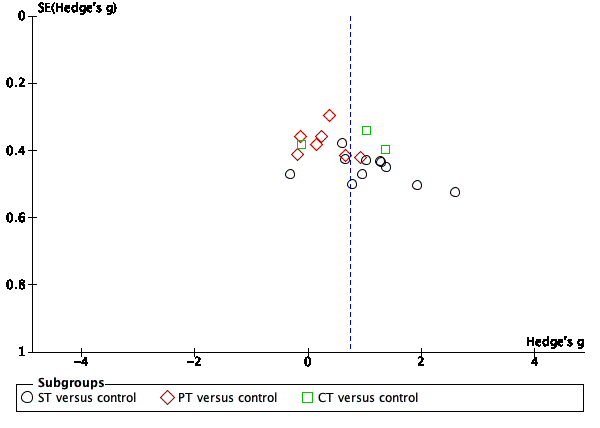

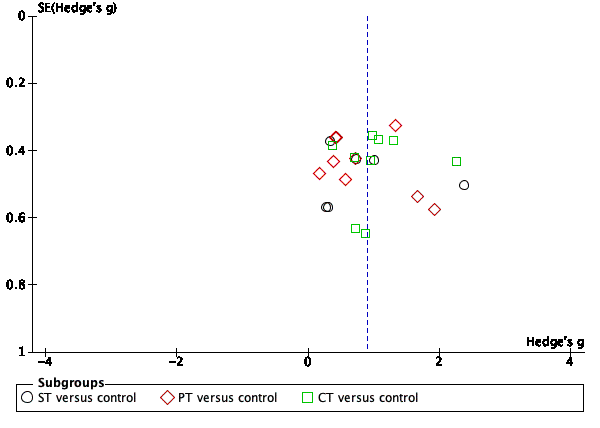

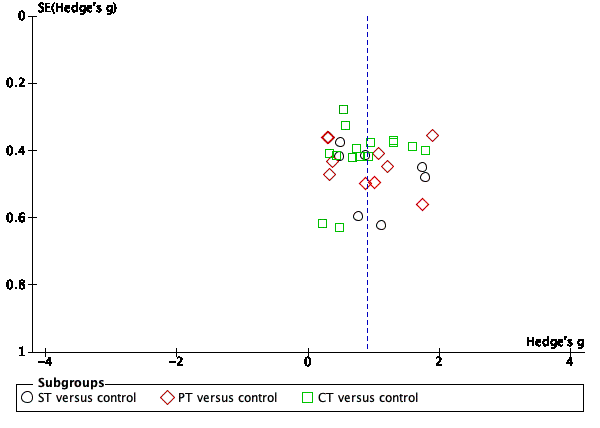

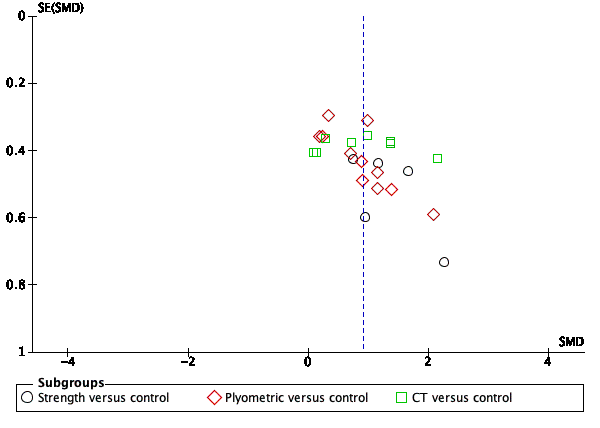

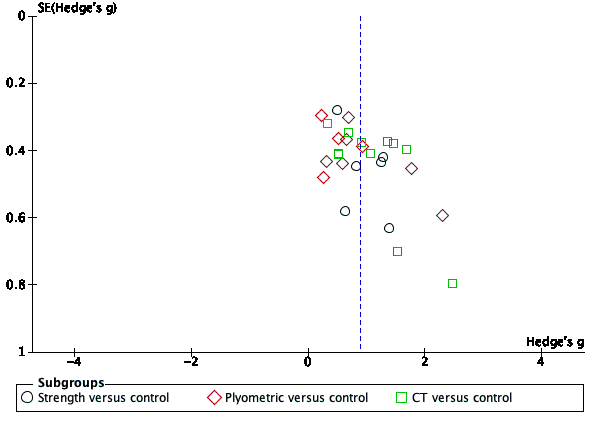

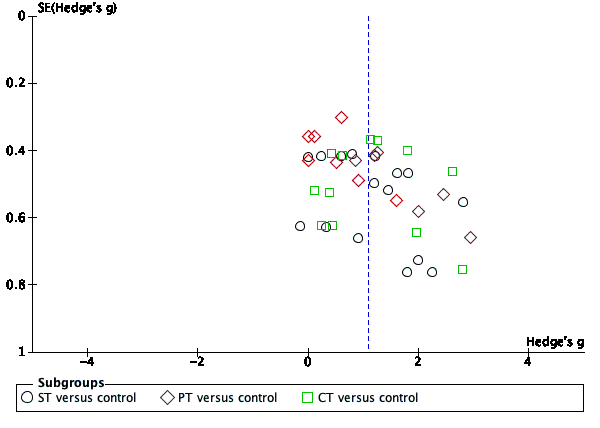

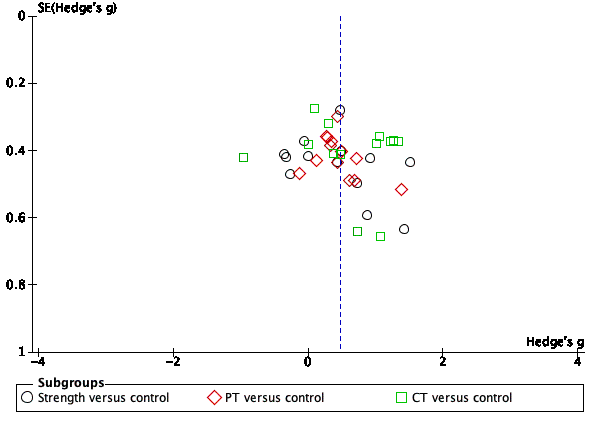


| **Supplementary Material 3:** Details of the exercises performed in each training intervention group and the outcomes measured in each of the included studies | | |
| --- | --- | --- |
| **Study** | **Training intervention** | **Exercises administered** |
| Abade et al. [88] | STG | STG: Bench press; Unilateral row; Barbell upright row; eccentric overload flywheel device (diagonal trunk rotation); front and lateral isometric planks.  STG1 (vertical): Back-half-squat  STG 2 (horizontal): Barbell hip thrusts  CON: Regular soccer training |
| Aloui et al. [89] | CTG  (PT + Short sprints) | CTG: Hurdle jumps; Lateral hurdle jumps; Bouncy strides; Single-leg hop jumps; Short sprint of 10-15 m  CON: Regular soccer training |
| Aloui et al. [92] | CTG  (PT + Short sprint with Change of direction) | CTG: Hurdle jumps; Bouncy strides and drop jump; 6 bouncy strides with change-of-direction.  CON: Regular soccer training |
| Aloui et al. [91] | CTG  (PT + Short sprint with Change of direction) | CTG: Hurdle jumps; Lateral hurdle jumps; Bouncy strides; Drop jumps and finished with a short change-of-direction sprint  CON: Regular soccer training |
| Aloui et al. [90] | CTG  (PT + Short sprint with Change of direction) | CTG: Hurdle jumps; Lateral hurdle jumps; Bouncy strides; Drop jumps and finished with a short change-of-direction sprint  CON: Regular soccer training |
| Alves et al. [64] | CTG  (ST + PT + Sprints) | CTG: 1RM squat at 90°; 1 set of 5-m high skipping; Calf extension exercise and perform 3 ball headers; Leg extension and jumped vertically to reach the highest point from the seated position on a stool; Drop jumps executing a soccer heading.  CON: Regular soccer training |
| Boraczyński et al. [65] | CTG  (ST + PT + Sprints) | CTG: High knee Back squat Triceps dips; Box jump Sit-ups; Vertical leg crunches; Butt kick; Plank punch; Incline push-ups; Russian twist; Jumping jacks; Jump squats; Burpee; Side skaters; Side plank; Jump tap; Line jumps; Standing calf raises; Walking lunge; Lunges; Inner thigh lifts; Mountain climber; Split squat; Lunge split jumps; 40-m repeated sprints  CON: Regular soccer training |
| Chelly et al. [26] | STG | STG: Back half squat  CON: Regular soccer training |
| Chtara et al. [66] | PTG | PTG: Countermovement jump; Line jump (standing distance jump); Drop jump + 1 step; Front to back cone hops; Lateral box jump push off; One leg distance jump + 1 step; Single-leg cone jumps front to back; Single-leg cone jumps side to side; Single-leg box Push off  CON: Regular soccer training |
| Saez de Villareal et al. [67] | CTG  (PT + Sprints) | CTG: 1/2 squat with jump; Skipping; Stride length; Sidelong jumps of 30-cm hurdle; Vertical jumps in 30-cm hurdle, and second support of triple jump; 10-m sprint + technical dribbling + shoot (the same shoot of test)  CON: Regular soccer training |
| Drouzas et al. [68] | PTG | PTG: Jumps in nine squares; Jumps over hurdles (10 cm) in four directions; Jumps in four directions after light signal; Jumps in four squares; Jumps over hurdles (15 cm) in four directions; Random jumps in nine squares; Side jumps; Standing jumps between two cones; Side jumps over a hurdle (10 cm); Lateral jumps from a balance mat; Lateral jumps over a cone; Lateral jumps over a hurdle (15 cm); Standing jumps on a balance mat  CON: Regular soccer training |
| Drury et al. [69] | STG | STG: Nordic hamstring exercise  CON: Regular soccer training along with passing drills |
| Ferrete et al. [70] | CTG  (ST + Sprints) | CTG: ¼ squat; Deep squat; Vertical jump; Obstacle jump; Weight displacement; Sprint exercises  CON: Regular soccer training |
| Franco-Marquez et al. [34] | CTG  (ST + PT) | CTG: Full squat; Countermovement jump; Step phase triple jump; Change of direction  CON: Regular soccer training |
| Hammami et al. [71] | STG, PTG | STG: Half-squats  PTG: 0.5-m hurdle jump; 0.6 m hurdle jump; 0.7-m hurdle jump  CON: Regular soccer training |
| Hammami et al. [72] | PTG | PTG: 3 hops to the right then 3 hops to the left and sprint 20 m; Lateral 0.3 m hurdle jumps (3 to left and 3 to right) and sprint 20 m; Horizontal jumps (three bell feet horizontal with the right leg follows from three bell feet horizontal with the left leg) and sprint 20 m; 6 × 0.4 m hurdle jumps and sprint 20 m  CON: Regular soccer training |
| Hammami et al. [73] | PTG | PTG: 0.5-m hurdle jump; 0.6 m hurdle jump; 0.6-m drop jump; 0.7-m hurdle jump; 0.7-m drop jump  CON: Regular soccer training |
| Hoshikawa et al. [74] | STG | STG: Elbow–toe; Elbow–heel; Side bridge; Modified 1-legged squat; Bent-knee push-up  CON: Regular soccer training |
| Keiner et al. [75] | STG, CTG  (PT + Sprints) | STG1: Parallel back squats; Deadlifts; Bench press; Pull ups; Sit-ups; Standing rows; Neck press; Crunches with additional weight  STG2: Mini band exercises: Lateral walk in a quarter squat stance; Forward backward zig-zag walk; Leg kicks; or body squats; Lateral walk with straight legs; Forward-backward tiptoe walk; Forward-backward walk in a parallel squat stance (monster walk); Prone kneeling exercise; Bridging with 1 leg to lift the pelvis; Planks while alternating lifting one foot from the ground; Push-ups in a TRX-band; Lateral bridging with alternating leg flexion and leg extension; Russian twist with a 3-kg medicine ball, rowing in a TRX-band  CTG: Squat; countermovement or drop jumps; 10-m to 30-m linear sprints, resisted sprints; Broad jump; Triple jump Change of direction sprints (e.g., up to 15 m with different degrees of change of directions)  CON: Regular soccer training. |
| Keiner et al. [28] | CTG  (ST + PT + Sprint + Core training) | CTG: Strength training: Lunges with additional weight Sit-ups; Overhead squat; Back squat; Front squat; Dead lift; Standing row; Bench press; Vertical jumps; Horizontal jumps; Lateral jumps; Linear sprints up to 15 m; Change-of-direction sprints up to 15 m; Medicine ball throws; Medicine ball jump-throws  CON: Regular soccer training |
| Keiner et al. [76] | STG | STG: Bench presses; Deadlifts; Neck presses; Exercises for the trunk and standing row  CON: Regular soccer training |
| Makhlouf et al. [77] | CTG  (PT + Balance training; PT + Agility training) | CTG: Countermovement jumps; Drop jumps + 1 step; Horizontal line jumps; Lateral hops; Ankle jumps; Single leg cone jumps front to back and side to side; Single leg maximal rebounding hops; Hurdle jumps; Drop from a low platform and perform ballistic -type push-ups or clapping push-ups  CON: Regular soccer training |
| Marques et al. [78] | CTG  (PT + Sprints) | CTG: 2-legged jumps; 2-legged jumps (knees bent); Short quick hops on one leg; 1-legged jumps as high as possible; Sprint from a standing position; Sprint from a lying position  CON: Regular soccer training |
| Michailidis et al. [29] | PTG | PTG: Single- and double-leg forward hops over hurdles, lateral hops over hurdles, and lateral shuffles over a box, skipping, and footwork (ladder drills). The second PT macrocycle consisted of footwork, skipping, single- and double-leg box jumps and relatively low-intensity depth jumps  CON: Regular soccer training |
| Negra et al. [79] | PTG | PTG: Vertical leap, whereas the second PT session focused on exercising horizontal jumps.  CON: Regular soccer training |
| Negra et al. [80] | STG | STG: Half squat  CON: Regular soccer training |
| Negra et al. [81] | STG, PTG | STG: Half squat  PTG: 2-footed ankle hop forward; hurdle jumps; and squat jump; Lateral plyometric training: lateral bound stabilization; lateral hurdle jumps; and double-leg zigzag  Additional exercises performed by both groups: abdominal curl; back extension  CON: Regular soccer training |
| Padron-Cabo et al. [82] | PTG | PTG: Rabbit hops; Two-foot side hops-down the side; Hopscotch; Snake jump; Backward hopscotch; Straddle hops; Skiers jumps; Two-foots hops-zigzag; Hopscotch variation  CON: Regular soccer training |
| Pena-Gonzalez et al. [83] | STG | STG: Semi-squat; Lunge; Side lateral lunge; Single-leg squat (right); Single-leg squat (left); Dead lift; Hip-thrust; Multi-jumping; Hip-rotation  CON: Regular soccer training |
| Ramirez-Campillo et al. [87] | PTG | PTG: Wall jump; Squat jump; Tuck jump; 180° jump (Broad jump and hold; Bounding in place; Bounding for distance; Single-leg hop and hold; Jump, jump, jump, vertical jump; Lunge jump; Crossover hop, hop, hop, stick (per leg) + Single-leg clock hop; 20-cm drop jumps  CON: Regular soccer training |
| Raya-Gonzalez et al. [84] | STG | STG: Flywheel resistance training  CON: Regular soccer training |
| Rodriguez-Rosell et al. [85] | CTG  (ST + PT) | CTG: Full squat; Countermovement jump; Change of direction; Sprint  CON: Regular soccer training |
| Szymanek-Pilarczyk [86] | PTG | PTG: Long, high, lateral and rotational jumps  CON: Regular soccer training |
| Wong et al. [35] | STG | STG: Bent-over row; Forward lunge; Upright row; Supine leg raise ™; Push up; Back half squat; Power clean; High pull; Weighted squat jump; Single-leg hop over hurdles  CON: Regular soccer training |
| **Abbreviations:** CON– control; CTG– combined training group; RM– repetition max; STG– strength training group; PTG– plyometric training group; STG– strength training group | | |
